# Optimal Short-Sighted Rules

**DOI:** 10.3389/fnins.2012.00129

**Published:** 2012-09-11

**Authors:** Sacha Bourgeois-Gironde

**Affiliations:** ^1^Laboratoire d’Economie Moderne, Université Paris 2Paris, France

**Keywords:** behavioral ecology, intertemporal choice, myopia, patch-paradigms, self-control

## Abstract

The aim of this paper is to assess the relevance of methodological transfers from behavioral ecology to experimental economics with respect to the elicitation of intertemporal preferences. More precisely our discussion will stem from the analysis of Stephens and Anderson’s (2001) seminal article. In their study with blue jays they document that foraging behavior typically implements short-sighted choice rules which are beneficial in the long run. Such long-term profitability of short-sighted behavior cannot be evidenced when using a self-control paradigm (one which contrasts in a binary way sooner smaller and later larger payoffs) but becomes apparent when ecological patch-paradigms (replicating economic situations in which the main trade-off consists in staying on a food patch or leaving for another patch) are implemented. We transfer this methodology in view of contrasting foraging strategies and self-control in human intertemporal choices.

## Introduction

**Figure 1:**
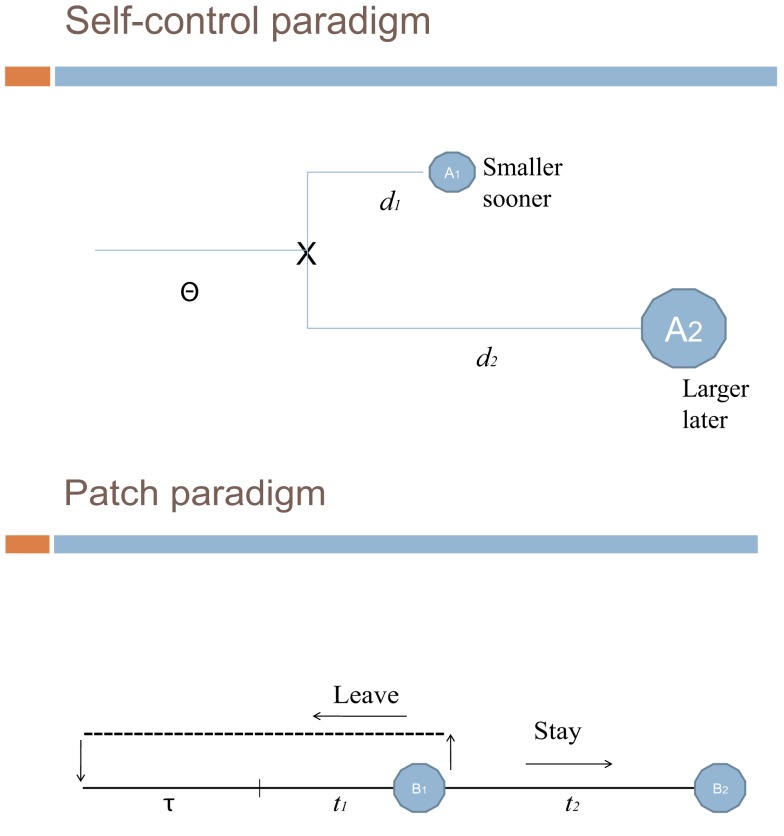
**Patch vs. binary-choice paradigms**.

The aim of this paper is to assess the relevance of methodological transfers from behavioral ecology to the neuroeconomics of intertemporal choices. More precisely, our discussion stems from the analysis of Stephens and Anderson’s (2001) seminal article. In their study with blue jays they report that foraging behavior typically implements short-sighted choice rules which are beneficial in the long run. Such long-term profitability of short-sighted behavior cannot be evidenced when using a self-control paradigm (one which contrasts in a binary way sooner smaller and later larger payoffs) but becomes apparent when ecological patch-paradigms (replicating economic situations in which the main trade-off consists in staying on a food patch or leaving for another patch) are implemented [see Figure 1]. Stephens and Anderson show that in certain situations (self-control settings) the immediate consequences of choice strongly influence animal behavior, while in other situations (stylized patch situations) animals adopt strategies apparently consistent with evolutionary models that emphasize the long-term fitness consequences of individual choices.

We schematize the two types of experimental paradigms and then address our target question as to know to what extent it is theoretically relevant to generalize them to issues recently addressed in the neuroeconomics of intertemporal choices. We defend a dual system underlying intertemporal choices, which is, however, distinct from McClure et al’s. (2004) view of a limbic system and a prefrontal system respectively encoding impatient and patient intertemporal choices. We rather focus on the contextual dependence/relevance of each of the two systems involved in that type of choices pleas in favor of the plausibility of optimal short-sighted behavior. This line of argument is briefly related to evolutionary considerations.

## Patches and Self-Control Paradigms in Intertemporal Choice Elicitation

### Animal self-control

Evolutionary theory predicts preferences for long-term decisions, if the issue is to guarantee the replication of a subset of genes making up an individual organism over a given temporal delay (until decay). Self-control paradigms are supposed to elicit those preferences at the individual level. In these settings animal have to wait for a time (*T*) and then have to make a binary choice between (1) a small-immediate reward (*t*_1_ → *G*_1_ → *p*) and a (2) large-delayed choice (*t*_2_ → *G*_2_; with *t*_2_ > *t*_1_ and *G*_2_ > *G*_1_), with a post-feeding delay (*p*) for one or both conditions. “Self-control” is defined as the case in which the subject waits for the large-delayed reward.

The long-term rate model predicts that in a self-control situation animals should choose the alternative 1 when the ratio of the first gain amount (*G*_1_) and the sum of the initial time (*T*), of the short delay (*t*_1_) and the post-feeding delay (*p*) is greater than the ratio of the second gain amount (*G*_2_) and the sum of the initial time (*T*) and the longer delay (*t*_2_), that is to say: *G*_1_/*T* + *t*_1_ + *p* > *G*_2_/*T* + *t*_2_. Long-sighted decisions involve temporal elements that play an important role in determining preferences but, as experimental evidence shows, animals treat these temporal elements in different ways:

- *Delays between choice and food delivery* strongly influence foraging preferences; in fact animals prefer shorter-delay even if the delayed amount is double (in some settings wherein self-control is particularly hard to maintain, among non-human animals only rhesus macaques seem to succeed; see Evans and Beran, 2007).
- *Post-feeding delay* yields virtually no effect on animal preferences, which discords with far-sighted models (Stephens et al., 2004).
- *Inter-trial intervals* (ITIs) make little effect on preferences, which again disagrees with far-sighted models (as shown, for instance, in Schultz, 2010).

As we can see self-control results contradict evolutionary models assuming long-term calculations. An obvious limitation of these models is their ability to accommodate small discounting effects, but their lack of account for long-term effects of systematic iterative short-sighted decisions. However, the potential optimality of iterative myopic behavior in the long run can be elicited by using the alternative patch-paradigm.

### Patches

In the patch-paradigm approach we define a “patch residence time” as the foraging duration spent by an animal on a particular area before it moves to another due to its observation or anticipation of local resources decrease (Stephens and Anderson, 2001). This approach relies on the prediction that patch residence times have an incidence on the long-term rate of food intake (Stephens and Krebs, 1986) and that foragers should spend more time in patches when travel times to a patch to another are longer. In fact travel time plays a role similar to the ITI but, contrary to what we observed with the self-control approach, in patch situations its effect is crucial. To the extent that foragers can choose between a small amount of food reachable in a short time and a large amount of food reachable in a longer time located on another patch, patch-paradigms implement a critical travel time cost. The contrast between staying on a patch and leaving that patch is this time expressed by a two-argument function that includes time and gain.

In spite of evidence to the effect that far-sighted foragers are sensitive to ITI, a question remains unaddressed: why in patch experiments long travel temporal intervals tends to induce animals to spend longer time to extract more food, while in self-control experiments ITI appears to have little effect? This question, as well as the apparent evidence that animals always adopt myopic strategies, has been tackled in an experiment where self-control and patch situations are parameterized as economically equivalent. In this experiment animals are trained to make as before (1) a binary choice between a small-immediate and a large-delayed gain (self-control), or (2) a choice between “leave” (small-immediate) and “stay” (large-delayed; patch-paradigm). The two situations are economically equivalent in so far as they present both the same conditions in terms of time and rewards (the same time/gain function as before). Since in this experiment the two situations are economically equivalent, if it is true that animals always adopt short-term strategies, the latter should be observable in both self-control and patch situations.

In order to establish the different patterns of choices in the self-control and patch-use contexts and because they had observed that ITI has an effect only in patch experiments, the authors tested each context at three distinct ITIs. To describe the differences in each combination (self-control/patch and ITI) they measured the effect for both 50 s and 5 s levels of delay-to-small reward. Results of the experiment demonstrated that when the delay-to-small reward (below abbreviated as DTS) was large (50 s) preferences of the blue jays were not affected by the ITI. However, when the delay-to-small was brief (5 s), the outcome was less tractable. In the control situation, the jays’ preference for large rewards decreased together with the ITI, while in the patch-use condition the subjects’ preference increased for the large reward together with the ITI. As predicted by evolutionary hypotheses about long-term fitness maximization patch-use situations revealed that jays favor large-delayed outcomes as ITI increased, but let us remind that in self-control cases, the conclusion was precisely the opposite.

To sum up, results show that:

- If DTS = 50 s then ITI has no effect on preference, but animals prefer large in the patch context.
- If DTS = 5 s then preferences for large increased with ITI in patch, while decreased in self-control. This shows an interaction between ITI and context in DTS.

The hypothesis proposed to explain these different behavioral patterns is that a single short-sighted behavioral rule underlies the approach to the different environments and their economic parameters. Self-control situations involving binary choices trigger a short-term rule that can be expressed simply as: “Choose 2 if *G*_2_/*t*_2_ > *G*_1_/*t*_1_.” This rule evidently disagrees with long-term maximization and ignores the potential impact of ITI in self-control contexts. However, the very same rule when applied in patch contexts may yield an optimal outcome, given that is these contexts the rule can be expressed as: “Choose 2 if [(*G*_2_ − *G*_1_)/(*t*_2_ − *t*_1_)] − [*G*_1_/(*T* + *t*_1_)] > 0.” The difference in terms of long run optimality of the rules across the two experimental paradigms can be easily explained if we pay attention to the fact that the difference in short-term rates is equivalent to the difference in long-term rates because in the patch context the short-term rule predicts sensitivity to T, the ITI term that constitute part of the key delay. Based on this result, it is possible to conclude that the short-term rule not only agrees, but significantly determines the difference in long-term rates, that is to say that the short-term rule explains the long-term maximization in the patch contrary to self-control situation.

## Optimal Foraging Strategies vs. Apparent Lack of Self-Control in Human Intertemporal Choice

Discounted utility theory (DUT) is the normative model used in order to account for intertemporal decisions. This model intends to capture the rationality of preferences over variably temporally located options under the joint criteria that those preferences are logically coherent, consistent over time and yield optimal payoffs. However, DUT has a restricted descriptive validity because it fails to capture more or less systematic violations of preferences temporal consistency. As neatly put by Kalenscher and Pennartz (2008): “Common difference and immediacy effects and the fact that preference reversals occur after deferring all choice alternatives into the future by the same interval, violate assumptions of consistent choice.” Foraging animals’ preferences might not essentially depend on the proportion of rewards and delays presented by alternative options but rather on the waiting time prior to the gains. The comparison of results for similar economic parameters over the two experimental paradigms demonstrates their incompatibility with an interpretation of foraging behavior in terms of sacrifice rather than maximization. It is not necessary to discard a short gain in order to maximize one’s fitness in the long run and short-term benefits may add up to optimal payoffs.

Let us note that these results in behavioral ecology are consistent with findings from McClure et al. (2004) study in which they observed that neural activities of the limbic system were greater for decisions involving choices between immediate and delayed rewards than for choices between only delayed rewards. Some specific neural mechanism is involved when short terms options are available. Yielding to immediate small rewards may be evolutionarily advantageous because once a small reward is consumed, it gets out of sight and temptation and the subject can pursue its longer-term goals. If gains are easy to grab, with very low opportunity costs, their immediate consumption may enhance the pursuit of life strategies by smothering tingling appetites. Our foraging ancestors may have developed this sense of taking advantages of small rewards as they presented themselves in their environments. Neural mechanisms dedicated to the valuation of those immediate rewards may thus have developed in order to deal properly with scarce and random resources. In our contemporary economic environments, this neural system may still prove itself useful. However this intuitive and evidence-based dual system approach defended by McClure and his colleagues is far from unanimously received.

Kable and Glimcher (2007) have certainly stated one of the most potent objections to the view that intertemporal choices are supported by a dual system such as the one McClure describes. More exactly, they contend that one general valuation system deals with different characteristics of economic options. It is a complex but single brain system that is, according to these authors, involved in intertemporal choice, in the sense that they make clear that the ventral striatum, the medial prefrontal cortex, and the posterior cingulated cortex tracks the subjective value of monetary rewards. Relative valuation, encoded by neural activities in the different areas constituting this whole system, corresponds to the selective manipulation of economic characteristics of the rewards. Namely, activity in those three main regions increases as the amount of the reward increases and decreases when the actualization delay of that reward increases. Kable and Glimcher thereby reduce intertemporal choice to option valuation according to different features processed single-handedly by one common neural valuation system.

We argue in favor of a midway between these two opposite neuroeconomic positions. The phenomenon of patience vs. impatience is robust but the current analyses of how such contrasted choices are encoded by the brain may miss the main point about the nature of these choices. Kable and Glimcher (2007), to our opinion, rightly point to the fact that as far as economic valuation is concerned, one neural system, with internally differentiated activities modulation, may be enough. The point is that economic valuation is not the only parameter (notwithstanding its relative complexity in terms of magnitude/delay trade-offs) at stake. Contextual evaluation in terms of probability of reward and richness of environment, being part of a broadened ecological approach of what intertemporal choices are like in natural and artificial economic settings, are essential parts of the nature of intertemporal choices and may motivate the adoption of a dual neural system in order to account for the contrast between apparent patience and impatience. But *pace* McClure et al. (2004) the dual system in question is not best explained in terms of those insufficiently contextualized behavioral denominations (patience/impatience) but rather in terms of optimal short-sighted behavior vs. optimal long-sighted behavior.

Kolling et al. (2012) have recently explored the neural mechanisms of foraging with human subjects. They demonstrate that humans can alternate between “stay” and “leave” strategies in multi-branched patch settings such as the ones we have schematized above. Humans process aptly the costs inherent to foraging choices. The contrast between such choices involves neural structures that partly (but only partly) overlap with the valuation system indicated by Kable and Glimcher (2007) and crosses over limbic and prefrontal systems respectively associated in McClure et al. (2004) to impatient and patient choices. “Stay or leave” choices in foraging settings involve distinct neural mechanisms in ventromedial prefrontal cortex (VPMC) and anterior cingulate cortex (ACC). VMPC activities are dedicated to a general valuation system, like reported by Kable and Glimcher but the ACC encodes the search cost and potential richness of alternative patching in the environment, which is something sufficiently neurally specific to this type of intertemporal choices setting. It seems to us then relevant to assess the optimality of short-sightedness and long-term choice behavior in terms of (i) the structure of economic settings (i.e., whether they present foraging potentialities or binary frames requiring self-control) and (ii) the correlation between the economic structure (here in terms of richness and search cost) and the contextual relevance of used behavioral rules within these structures.

## Conclusion

Modern economic environments are labile and complex and the propensity to accept small rewards may be optimal in the face of the opportunity costs of more sophisticated strategies. It is also possible that the incorporation of long-term plans and self-projections in the far future into present decisions is more evolutionary recent than the tendency to accept immediate gratifications. From that evolutionary perspective, the preference of small-immediate rewards over larger future ones is not the sign of our irrationality, but may rather reflect the conflict between two evolved rational rules: the incremental pursuit of long-term goals and the maximization of low cost immediate rewards. Patch-paradigms used in behavioral ecology precisely demonstrate the compatibility and optimal coincidence of these potentially jointly evolutionarily selected behavioral rules. The apparent conflict shown by opposed behavioral data over self-control and patch-paradigms is solved if one considers, on the one hand, that aggregate immediate gains may add up to maximizing long-term fitness and, on the other hand, that predefined long-term goals are endogenously modified by actually made choices.

Monterosso and Ainslie (1999) note that “people and less cognitively sophisticated animals do not differ in the hyperbolic form of their discount curves.” Some researchers (e.g., Herrnstein, 1997; Rachlin, 2000) hold the view that hyperbolic time discounting is effectively “hardwired” into our evolutionary apparatus. However, time discounting of humans and other animals may also rely on qualitatively different mechanisms. While both humans and animals discount the future at dramatically different rates, both humans and animals display a common pattern of time discounting commonly referred to as “hyperbolic time discounting.” However, they believe that while such findings do not rule out the possibility that humans and animals discount the future similarly, the quantitative discontinuity is indicative of a qualitative discontinuity. It is not that clear that discounting of humans and other animals relies on qualitatively different mechanisms even though, recent neuroeconomic studies (such as McClure et al’s., 2004) tended to support that, specifically, human time discounting reflects the operation of two fundamentally different systems, one that heavily values the present and cares little about the future (which we share with other animals), and another that discounts outcomes more consistently across time (which is uniquely human). More extended and systematic comparisons between foraging patches and self-control paradigms among human subjects could help to revisit this view.

Microeconomics research has seldom considered animals as possible research subjects, but in recent years evolutionary theories of human and animal decision making might show how such a transfer of methodologies and theoretical goals could be fruitful (Kalenscher and van Wingerden, 2011). Starting from evolutionary considerations we can understand how the uncovering of choice mechanisms in animals and their neural substrates may help understand human intertemporal choice behavior. Moreover, economic theories and ecological models show remarkable similarities in their assumptions and implications (Stephens and Krebs, 1986). Although the decision rules used by modern humans take place in a different context, they evolved in a similar context and they may actually be maladaptive today to some extent (Kahneman and Tversky, 1996). But it can also be envisioned that Stephens and Anderson (2001) provide a useful tool to understand that modern humans’ decision strategies are optimally adapted to the sequential foreground/background environment faced by foragers, but at the same time they may fail to produce an optimal outcome in a “modern” binary choice environments.

## Conflict of Interest Statement

The author declares that the research was conducted in the absence of any commercial or financial relationships that could be construed as a potential conflict of interest.
